# Supplementary material for: Effects of Factors Influencing Scar Formation on the Scar Microbiome in Patients with Burns
Source: Int J Mol Sci. 2023 Nov 6;24(21):15991. doi: 10.3390/ijms242115991 (PMC10648024; doi:10.3390/ijms242115991)
Supplement: Supplementary file 1 [file ijms-24-15991-s001.zip › ijms-2650766-supplementary.pdf]

Supplementary materials

Effects of Factors Influencing Scar Formation on the Scar Microbiome in Patients with Burns

Yeongyun Jung [1], Hui Song Cui [1], Eun Kyung Lee [1], So Young Joo [2], Cheong Hoon Seo [2] and Yoon Soo Cho [2,*]

[1] Burn Institute, Hangang Sacred Heart Hospital, Hallym University College of Medicine, Seoul 07247, Republic of Korea; jyg1076@hallym.ac.kr (Y.J.); bioeast007@naver.com (H.S.C.); eunlee0617@gmail.com (E.K.L.)

[2] Department of Rehabilitation Medicine, Hangang Sacred Heart Hospital, Hallym University College of Medicine, Seoul 07247, Republic of Korea; anyany98@gmail.com (S.Y.J.); chseomd@gmail.com (C.H.S.)

* Correspondence: yschorm@hallym.ac.kr; Tel.: +82-02-2639-5739; Fax: +82-02-2633-7571

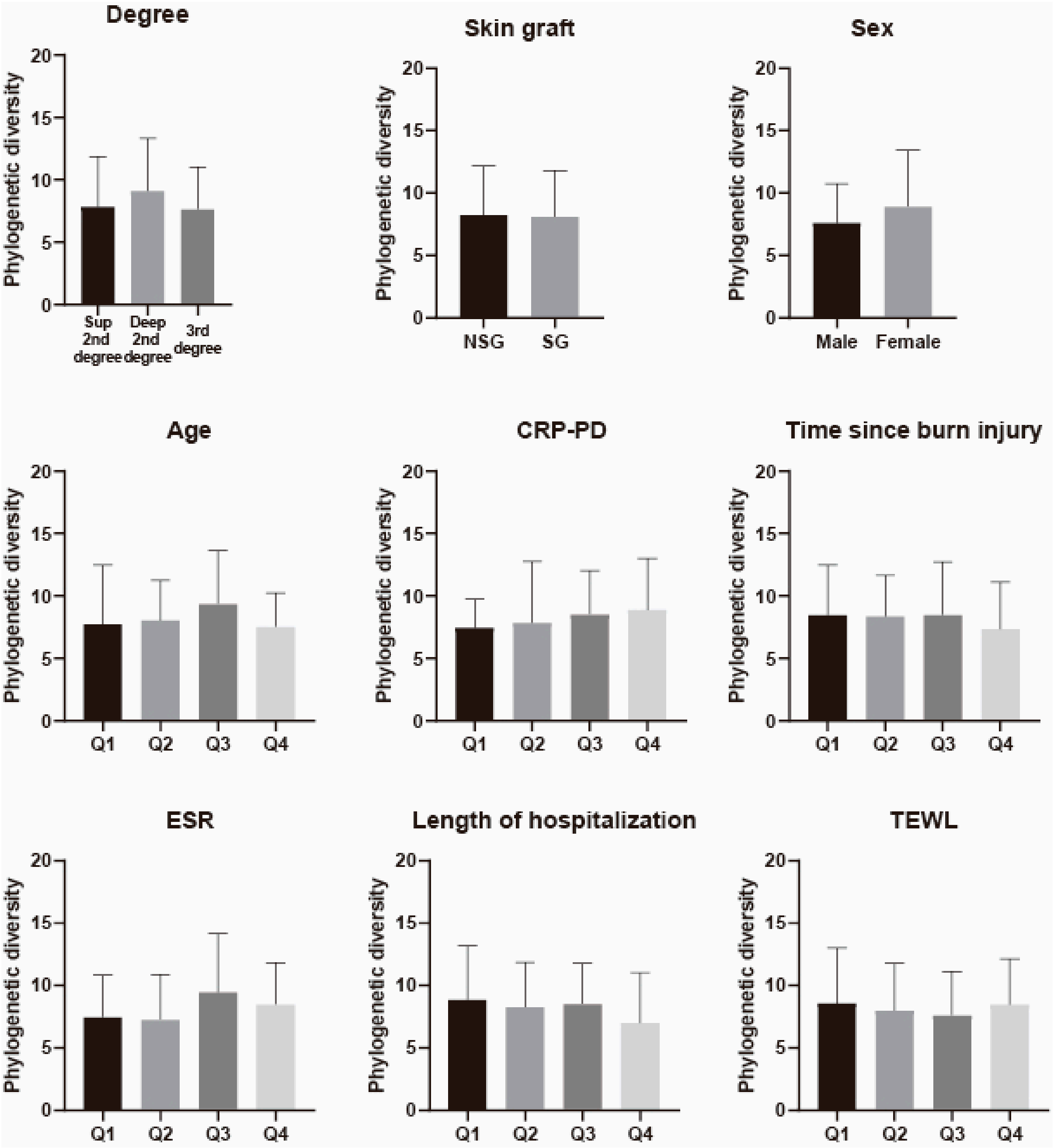

Supplementary Figure S1. Comparison of the alpha diversity indexes of skin microbiota according to factors influencing hypertrophic scar formation. Values are expressed as means ± standard deviation.

Supplementary Table S1. Biomechanical characteristics of participants and burn scars

Variables		Patients with burns (*n* = 40)
**Age, years**		46.15 ± 11.68
**Sex**	Male	23 (57.50%)
	Female	17 (42.50%)
**Degree**	Superficial 2nd degree	9 (22.50%)
	Deep 2nd degree	13 (32.50%)
	3rd degree	18 (45.00%)
**Skin graft**	Yes	20 (50.00%)
	No	20 (50.00%)
**TBSA burned, %**		14.00 (6.00, 27.00)
**ESR, mm/H**		11.00 (5.00, 15.25)
**CRP, mg/L**		0.98 (0.51, 2.02)
**Length of hospitalization, days**		22.50 (15.50, 39.75)
**ICU admission**	Yes	10 (25.00%)
	No	30 (75.00%)
**Time after injury, days**		100.48 ± 66.04
**Variables**		**Scar samples (n = 80)**
**Scar type**	Center of scar	40 (50.00%)
	Scar margin	40 (50.00%)
**Itch NRS**		0.00 (0.00, 4.00)
**Thickness, mm**		0.02 (0.01, 0.15)
**Melanin, AU**		156.74 ± 67.04
**Erythema, AU**		301.31 ± 141.94
**TEWL, g/m^{2}h**		11.77 ± 4.05
**Skin hydration, AU**		45.97 ± 14.94

Data are presented as mean ± standard deviation or median (25th percentile, 75th percentile) for continuous variables or numbers (%) for categorical variables. Abbreviations: TBSA, total body surface area; ESR, erythrocyte sedimentation rate; CRP, C-reactive protein; ICU, Intensive care unit; NRS, numerical rating scale; TEWL, transepidermal water loss; AU, arbitrary unit.

Supplementary Table S2. Biomechanical characteristics of participants and scars according to the subgroups

Variables		Group (patients with burns)								
		Sex		*P*-value	Skin graft		*P*-value	ICU admission		*P*-value
		Male (*n* = 23)	Female (*n* = 17)		Yes (*n* = 20)	No (*n* = 20)		Yes (*n* = 10)	No (*n* = 30)	
**Age, years**		44.96 ± 11.94	47.76 ± 11.85	0.468	47.30 ± 11.13	45.00 ± 12.67	0.597	47.80 ± 10.50	45.60 ± 12.36	0.650
**Sex**	Male	23 (100.00%)	0 (0.00%)	<0.001	11 (55.00%)	12 (60.00%)	0.749	8 (80.00%)	15 (50.00%)	0.097
	Female	0 (0.00%)	23 (100.00%)		9 (45.00%)	8 (40.00%)		2 (20.00%)	15 (50.00%)	
**Degree**	Superficial 2nd degree	5 (21.74%)	4 (23.53%)	0.938	0 (0.00%)	9 (45.00%)	<0.001	2 (20.00%)	7 (23.33%)	0.138
	Deep 2nd degree	8 (34.78%)	5 (29.41%)		2 (10.00%)	11 (55.00%)		1 (10.00%)	12 (40.00%)	
	3rd degree	10 (43.48%)	8 (47.06%)		18 (90.00%)	0 (0.00%)		7 (70.00%)	11 (36.67%)	
**Skin graft**	Yes	11 (47.83%)	9 (52.94%)	0.102	20 (100.00%)	0 (0.00%)	<0.001	7 (70.00%)	13 (43.33%)	0.144
	No	12 (52.17%)	8 (47.06%)		0 (0.00%)	20 (100.00%)		3 (30.00%)	17 (56.67%)	
**TBSA burned, %**		20.48 ± 15.87	16.06 ± 14.35	0.305	25.05 ± 17.44	12.15 ± 9.12	0.020	36.30 ± 15.80	12.70 ± 9.47	<0.001
**ESR, mm/H**		10.70 ± 9.17	12.65 ± 6.33	0.170	13.20 ± 7.92	9.85 ± 8.04	0.125	13.30 ± 9.06	10.93 ± 7.77	0.472
**CRP, mg/L**		2.37 ± 3.32	2.03 ± 2.87	0.299	3.07 ± 3.75	1.38 ± 2.06	0.147	2.27 ± 2.40	2.21 ± 3.35	0.170
**Length of hospitalization, days**		26.65 ± 13.82	27.18 ± 25.49	0.537	36.65 ± 20.28	17.10 ± 12.50	0.002	40.40 ± 16.01	22.37 ± 18.44	0.007
**ICU admission**	Yes	8 (34.78%)	2 (11.76%)	2.762	7 (35.00%)	3 (15.00%)	0.144	10 (100.00%)	0 (0.00%)	<0.001
	No	15 (65.22%)	15 (88.24%)		13 (65.00%)	17 (85.00%)		0 (0.00%)	30 (100.00%)	
**Time after injury, days**		96.91 ± 67.28	105.30 ± 68.09	0.593	130.40 ± 74.01	70.60 ± 42.71	0.003	153.40 ± 76.87	82.83 ± 53.79	0.005

Variables		Group (burn scars)								
		Sex		*P*-value	Skin graft		*P*-value	ICU admission		*P*-value
		Male (*n* = 46)	Female (*n* = 34)		Yes (*n* = 40)	No (*n* = 40)		Yes (*n* = 20)	No (*n* = 60)	
**Scar type**	Center of scar	23 (50.00%)	17 (50.00%)	>0.999	20 (50.00%)	20 (50.00%)	>0.999	10 (50.00%)	30 (50.00%)	>0.999
	Scar margin	23 (50.00%)	17 (50.00%)		20 (50.00%)	20 (50.00%)		10 (50.00%)	30 (50.00%)	
**Itch NRS**		2.00 ± 2.58	2.61 ± 3.35	0.541	2.88 ± 3.35	1.65 ± 2..33	0.155	2.55 ± 3.60	2.16 ± 2.66	0.984
**Thickness, mm**		0.10 ± 0.16	0.12 ± 0.18	0.787	0.17 ± 0.20	0.05 ± 0.10	0.039	0.15 ± 0.21	0.10 ± 0.15	0.486
**Melanin, AU**		161.30 ± 61.44	150.50 ± 75.35	0.125	152.40 ± 62.28	161.10 ± 72.81	0.802	149.40 ± 68.90	159.50 ± 67.30	0.754
**Erythema, AU**		313.90 ± 136.60	284.30 ± 151.30	0.238	313.10 ± 145.40	289.50 ± 141.10	0.459	287.80 ± 140.30	306.40 ± 144.60	0.628
**TEWL, g/m²h**		12.70 ± 3.76	10.52 ± 4.20	0.003	12.41 ± 4.36	11.14 ± 3.71	0.169	11.55 ± 3.69	11.86 ± 4.24	0.917
**Skin hydration, AU**		47.28 ± 14.13	44.20 ± 16.22	0.623	45.00 ± 16.13	46.95 ± 14.00	0.675	51.28 ± 15.27	43.96 ± 14.58	0.064

Data are presented as mean ± standard deviation or median (25th percentile, 75th percentile) for continuous variables or numbers (%) for categorical variables. Abbreviations: TBSA, total body surface area; ESR, erythrocyte sedimentation rate; CRP, C-reactive protein; ICU, Intensive care unit; NRS, numerical rating scale; TEWL, transepidermal water loss; AU, arbitrary unit.
